# Video Display Operator Complaints: A 10-Year Follow-Up of Visual Fatigue and Refractive Disorders

**DOI:** 10.3390/ijerph16142501

**Published:** 2019-07-13

**Authors:** Francesca Larese Filon, Anna Drusian, Federico Ronchese, Corrado Negro

**Affiliations:** Unit of Occupational Medicine, University of Trieste, 34100 Trieste, Italy

**Keywords:** follow-up, video display terminal, visual fatigue, refractive disorders

## Abstract

Visual fatigue and discomfort are very common complaints for video display operators (VDTs). The aim of our study was to study work-related visual symptoms in relation to refractive disorders and psychosocial factors in 3054 public employees by way of follow-ups for 10 years with periodic medical examinations with eye evaluation in the period 2000–2009. Factors related to visual fatigue were evaluated in the follow-up using generalized equation estimation. Visual fatigue was very common in VDT operators (64.03%). During the follow-up, no relationship between visual fatigue and age, sex, seniority of work, visual acuity and refractory disorders was found. Visual fatigue was significantly associated with anxiety perception in a dose-related matter (odds ratio (OR) 7.40, confidence interval (CI) 95% 1.77–31.3), psychosocial factors (OR 1.03, CI 95% 1.01–1.07), use of lenses (OR 1.34, CI 95% 1.09–1.64) and time of VDT usage (OR 1.27, CI 95% 1.04–1.53). This study confirmed that visual fatigue is common in VDT users and is related to anxiety perception, time of VDT usage, use of lenses and stress. No relationship was found between visual fatigue and refractory disorders or visual acuity.

## 1. Introduction

Workers that spend many hours on video display terminals (VDTs) can report visual fatigue and discomfort related to their work environment as well as vision defects, psychosocial stress and anxiety perception [1,2,3,4]. Many authors have studied musculoskeletal disorders in VDT operators that increased due to non-ergonomic workstations and postural demands [5,6], but there are, to our knowledge, no long-term follow-up studies that have analyzed visual fatigue and related factors.

Eye symptoms are very common in VDT users, and the American Optometric Association has started to refer to computer vision syndrome (CVS) as the combination of eye and vision problems associated with the use of computers. This derives from insufficient visual capabilities to perform the computer task comfortably. CVS is characterized by itching, redness, burning, eye tearing, headache, double vision, eye strain and blurred vision [1]. The prevalence of CVS increases significantly in individuals who spend more than 4 h daily working on VDT [4]. However, personal, environmental and ergonomic factors are also relevant as well as the long-term use of VDTs after work hours and mobile phones [7].

Visual fatigue is a major complaint in subjects with CVS and is characterized by weakness of the eyes, usually accompanied by headache and dimming of vision—symptoms that are transitory and recede with rest [4,8,9,10,11,12,13]. Eye fatigue appears because of the unconscious muscular effort of the eyes when preventive measures are not taken. Eye fatigue can be induced by repeated activation/deactivation of the ocular muscles [14] or by prolonged accommodative responses to similar focal distances [15]. The use of VDT leads to a reduction in the amplitude of accommodation with an increase in exophoria [16]; however, these alterations are temporary changes with no long-term permanent effect [1]. Close work can also induce transient myopia, and Luberto et al. [17] in 1989 suggested that the use of a temporary myopic shift can be a reliable objective assessment tool for VDT-related visual fatigue.

The subjective asthenopic symptoms, however, can also be related to environmental factors, such as lighting, screen resolution and work arrangements [18]. The surrounding illuminance causes greater changes in visual function with the reduction of amplitude of accommodation; also, the increase in illuminance has a negative effect on reaction time. Moreover, red and green-colored lights produced more visual fatigue compared to white and blue-colored lights. Janosik and Grzesik [19] in 2003 recommended illumination higher than 200 lux. The presence of reflections on the skin causes confusion, with multiple attempts required at focused reading on VDT and the need for additional accommodation responses. Working hours on VDTs without breaks can increase visual fatigue, and a relative humidity below 40% and high temperature can cause ocular discomfort and dry eye syndrome [10].

Visual fatigue prevalence ranges between 19.6% [20] and 72.1% [21] in office workers. The wide range of prevalence depends on the criteria used to define symptoms and methods used [20].

In addition to ergonomic and environmental factors [7,22,23], some research has focused on psychological factors as causative factors of CVS. Office work is sedentary and requires less physical energy than other jobs, but needs more mental attention and cognitive processes, sometimes with constant work pressure and at others with low levels of autonomy and few decision-making possibilities that can cause stress [24].

Visual fatigue and stress are common in VDT users [25,26,27] and are sometimes associated with musculoskeletal disorders [28], but research is lacking in terms of long-term follow-up. The aim of our study was to evaluate these symptoms in a large group of VDT users in 10-year follow-up, with the final aim of suggesting interventions which are useful to increase wellness in VDT workers.

## 2. Materials and Methods

We evaluated 3054 computer operators working with VDTs for more than 20 h per week in different public offices in Trieste (Italy) that underwent periodical health surveillance in the Clinical Unit of Occupational Medicine from 2000 to 2009. Two thousand two hundred and eight workers completed the follow-up with 3 or 4 medical examinations according to age: workers underwent a periodic medical evaluation with eye examination, as required by Italian law (every 5 years for workers <50 years-old and every 2 years for those ≥50 years-old). Lenses in use were defined using a lensmeter (Essilor Instruments CL60, Thornbury, Bristol, UK). Myopia was defined when >−0.25 dioptres (D). Hyperopia and presbyopia were defined when >0.25D and astigmatism was defined when >0.25D. Visual acuity using lenses was evaluated for far, near and intermediate distances (57 cm) using the Vision test (Essilor Instruments, Thornbury, Bristol, UK).

During the medical examinations, workers filled in a standardized questionnaire, divided into three sections. (1) The first part comprised 59 questions concerning personal data, years of use of a VDT, hours of use per day, computer programs used, screen size and type, work breaks, interruptions during VDT work, work environment in terms of lighting, glare on the screen, distance between the computer and operator, working desk, environmental noise and temperature; (2) the second part analyzed workers’ life and health, marital status, schooling, sport, smoking habit, coffee consumption, eye illnesses, use of lenses, eye examinations, and joint diseases; (3) the third part investigated the frequency of eye-symptoms related to VDT use: fatigue, redness, burning, tearing, headache, photophobia, far and near blurred vision, myodesopsia, double vision, and eye drop application—defined as never, sometimes, and often in relation to VDTs or not related to VDTs; (4) anxiety perception was defined as never, sometimes, and often in relation to VDTs or not related to VDTs (the question asked was “do you feel anxious during work with VDTs? “, and possible answers were “never”, “sometimes”, “often” or “not related to VDT use”); (5) psychosocial factors were evaluated using a simplified questionnaire that investigated discomfort (1—yes or 0—no) during work at VDTs regarding the following aspects: overcrowding, relationship with users, relationship with colleagues, work condition, responsibility, repetitiveness, excessive concentration, excessive attention, low possibility of correcting errors, frequent interruption during work, computer slowness, excessive variability of work tasks, low control of work tasks, slow cadence of work tasks, and fast cadence of work tasks. Psychosocial factors were summarized as the sum of all answers. All subjects signed an informed consent, and data were analyzed anonymously. Periodical medical surveillance is compulsory in Italy under the law (81/2008) and, according to Italian rules, the Local Ethical Committee was informed about the study.

Data were collected using the program Excel for Windows, and statistical analyses were performed using the STATA program (StataCorp, 4905 Lakeway Drive, College Station, Texas, US). Continuous data were compared using the t-Student test and t-pair test for repeated measures; categorical data were compared with the chi-square test and with Mc Nemar test for repeated measures. Relationships between visual fatigue, occupational data and other symptoms were evaluated with Pearson correlation and univariate logistic regression. Significant findings were studied with multivariate logistic regression. The relationship between visual fatigue and potentially associated variables during follow-up was evaluated using generalized estimating equations (GEE) that permit the study of the same subject over time. Statistical significance was settled at *p* < 0.05.

## 3. Results

**Figure 1:**
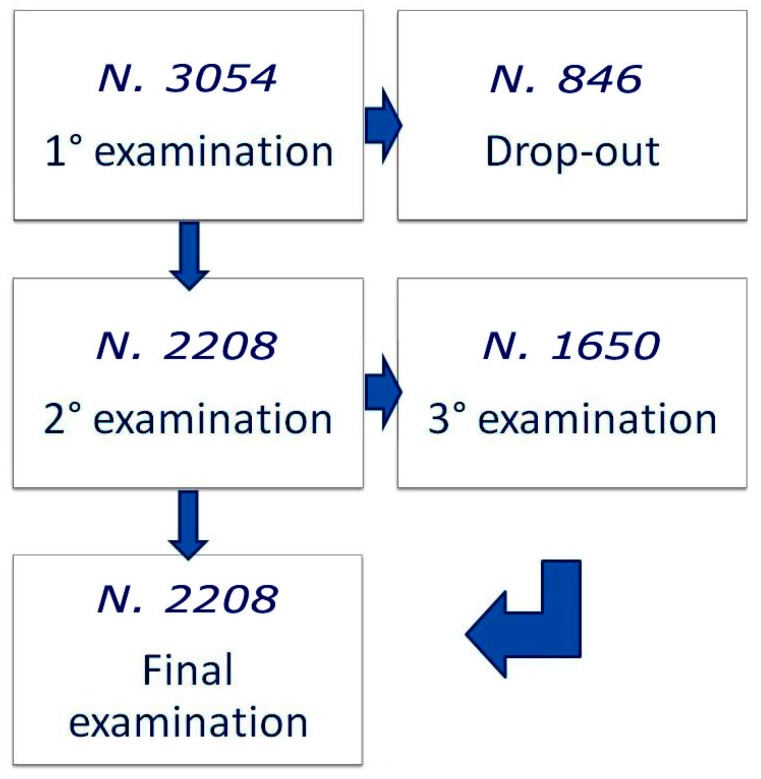
Design of the study.

Figure 1 reports the study design: 3054 subjects underwent the first control, and 2208 completed the follow-up with 3 or 4 controls, as required by Italian law, depending on age.

The characteristics of the workers involved in the study are reported in Table 1. Gender is represented in similar proportions, and age increased obviously from the first control (36.7 ± 10.5 years) to the last one (46.5 ± 9.3 years) with *p* < 0.005. A similar trend was found for work seniority starting at 7.4 ± 6.7 years in the first control and reaching 14.5 ± 7.4 in the last follow-up (*p* < 0.001). The time of VDT use was around 5 h per day in the period considered. Refractive defects in workers over time are shown in the same table. Myopia was quite stable in terms of years; astigmatism ranged from 37.14% to 42.97%. Presbyopia increased according to the age of people (from 36.82% in the first examination to 52.26% in the last one (*p* < 0.001). Hyperopia grew from 7.5% in the first examination to 9.94% at the intermediate follow-up and to 11.59% at the last control (*p* < 0.001).

The use of lenses was very common during the follow-up, with more than 80% of people using lenses.

The most common eye symptom was visual fatigue, followed by ocular redness, burning and tearing. In general, eye symptoms increased during follow up, reaching statistical significance for visual fatigue (58.4% to 64%, *p* < 0.001), ocular redness (28.9% to 35.2%, *p* < 0.001), and near blurred vision (8.7% to 12.9%, *p* < 0.01). Headache and anxiety were reported in similar figures during the follow-up (12% to 14% and 3.5% to 4.2%), respectively.

**Figure 2:**
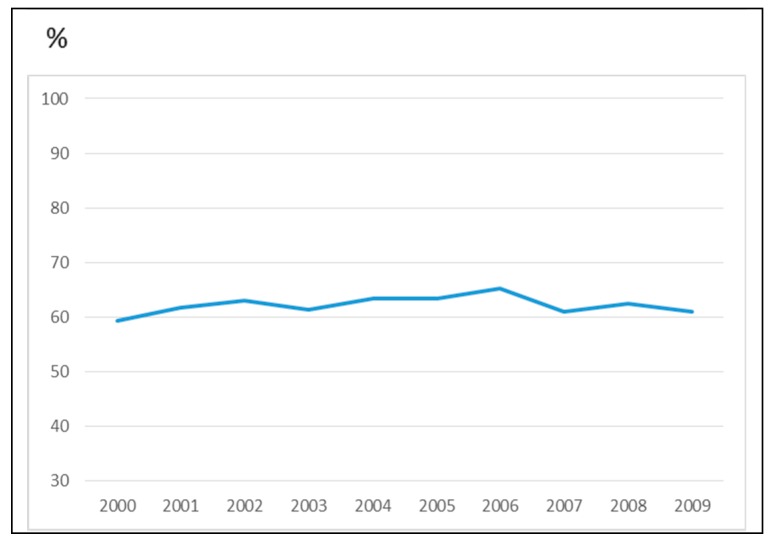
Percentages of visual fatigue in terms of years.

We evaluated visual fatigue in terms of years (Figure 2) to verify the improvement of symptoms in relation to the better quality of the screens; however, no difference was shown and no decrease in symptoms was reported.

Visual fatigue, analyzed using Pearson’s correlation, was not associated to far, near, or intermediate vision acuity. Univariate analysis confirmed this with values of OR = 1.1; 95% CI 0.98–1.24; OR = 1.02; 95%CI 0.97–1.07; OR = 1.06; CI95% 0.96–1.2, respectively. Visual fatigue was studied in relationship with environmental and ergonomic factors such as monitor characteristics, work place illumination, reflections on the screen, contrast, and incorrect distances from the computer using Pearson’s correlation (Table 2).

All factors except natural light were significantly related with visual fatigue. The relationships between visual fatigue and psychosocial variables are reported in Table 3.

All psychosocial variables were significantly related each other. Factors associated to visual fatigue in the first and last examinations were evaluated using univariate logistic regression (Table 4).

In the first examination, females reported more visual symptoms, but no gender effect was shown in the last examination. Age was inversely related to visual fatigue, and seniority of work was also demonstrated to be a protective factor in the last control. Visual fatigue increased significantly in relation to VDT working hours and use of lenses. Visual acuity at near, intermediate and far distances was not related to ocular symptoms and, when analyzing refractive disorders, hyperopia increased ocular symptoms, but only in the first examination. To evaluate the role of different factors, we analyzed visual fatigue using the multivariate logistic regression in the first control (Table 5).

Environmental factors were summarized in one variable that did not include natural light, which were not related with visual symptoms. Psychosocial factors were summarized in one item obtained by the sum of single items, and anxiety perception was analyzed alone as never, sometimes, and often both in relation with VDT use and not being work-related. In multivariate analysis, visual fatigue was not related to age, sex or work seniority, while symptoms increased with anxiety perception in a dose-related matter with OR 2.62 (CI 95% 1.56–4.4) and OR 4.97 (CI 95% 1.48–6.84) for “sometimes” or “often”, respectively. VDT working hours, use of lenses, hyperopia and psychosocial factors were significantly related to visual fatigue.

To analyze factors related to visual fatigue in the follow-up, we used the generalized estimating equation (GEE) as shown in Table 6.

Age was inversely associated with visual fatigue, and no relationship was found with seniority of work and gender. Anxiety perception was strongly associated with visual fatigue in a dose-related matter with OR 2.69 (CI 95% 1.57–4.61) for “sometimes” feeling anxious during VDT use and OR 7.10 (CI 95% 1.77–31.03) for “often” feeling anxious during VDT use. A significant relationship was demonstrated also with hours of work with VDTs (OR 1.27; CI 95% 1.04–1.53), use of lenses (OR 1.34, CI 95% 1.09–1.64), environmental factors (OR 1.02; CI 95% 1.01–1.06) and psychosocial factors (OR 1.03; CI 95% 1.01–1.07).

## 4. Discussion

Many epidemiological studies on VDT users have reported the presence of computer vision syndrome [29], and our study confirmed this in a long-term follow-up. However, the longitudinal characteristics of our study permitted us to analyze factors related to visual symptoms in a more comprehensive way, finding that anxiety perception was the most important factor, followed by the time of VDT usage, the use of lenses, environmental and psychosocial factors. No associations were found with seniority of work and visual acuity. Age appeared to be inversely related to visual symptoms. Visual fatigue grew with increasing working hours in a poorly controlled environment with light disturbance [8,30,31], and VDT work could worsen visual problems [18,32,33,34] in the short term [35,36]. However, there was no relationship between refractive disorders and VDT use in a 4-year follow-up [37], and visual fatigue decreased significantly with age, as also shown in other studies [38], probably because older workers know better how to manage visual discomfort, despite the higher prevalence of presbyopia. Dry-eye syndrome can influence visual fatigue, and the use of contact lenses can increase ocular symptoms [39,40]. The wearing of contact lenses causes a reduction of blink amplitude and tear film instability with Meibomian gland atrophy with tear alteration with an increasing of dry-eye symptoms. The desiccation of the contact lens surfaces causes irregular refraction with visual discomfort [40].

Regarding refractive disorders, myopia prevalence did not increase in follow-up, while presbyopia and hyperopia were clearly age-related and astigmatism increased in small amounts during follow-up. We found visual defects to be higher than those in the literature [41] because we considered any view change starting from 0.25 dioptres—thus, with a lower cut-off—whereas in a European Eye Consortium study, myopia was defined as ≥ −0.75 dioptres, with hyperopia and presbyopia ≥ 1D. However, myopia prevalence did not increase during the follow-up, and we failed to find an association between myopia and work seniority. Overall, no relationship was found between visual fatigue, refractive disorders and visual acuity with lenses during the follow-up, while in the first examination, only hyperopia was significantly associated with visual complaints.

Visual fatigue was also related to environmental and ergonomic factors such as monitor characteristics and workplace illumination, in accordance with the literature [42,43,44]. Poor illumination, the presence of contrast, light flickering and reflections on the screen, poor-resolution images and an incorrect distance to the computer contributed to eye-symptom development. Environmental factors were associated with visual fatigue in our study as well, despite the fact that, in the last 10 years, the introduction of better flat video screens has resulted in an improvement of image quality.

Psychosocial factors were associated with visual fatigue. They are, in addiction to ergonomic factors, the basis of the occurrence of somatic disorders (such as headache and insomnia), generalized tension and stress [45,46,47]. All psychological variables were positively related to each other, confirming the fundamental role of perception of these factors in predicting the onset of symptoms in the workplace.

Some factors were more related to visual fatigue than others. Work in crowded places, having problems with colleagues, performing tasks that require too much attention and concentration, and having little control over decisions about work, intensity of work have relevant impacts on the occurrence of visual symptoms. Other important factors are job position and the repetitiveness of working duties.

Another important issue that must be considered in people using VDTs is motion sickness, which has been studied mainly in video game users [48] or in people watching 3D movies [49] but which can be present also in workers using mobile devices [50,51]. Visually-induced motion sickness experienced in a 3D immersive virtual environment is one of the crucial aspects that limits the widespread use of virtual reality [52] and can contribute to visual fatigue in VDT workers. However, in our study—a long-term follow-up—we did not consider this symptom, because the widespread use of mobile devices started after the start of our project. This can be considered a limitation of our study; however, we tried to focus on visual fatigue and stress, which were the most important symptoms in VDT users, together with musculoskeletal symptoms.

Our study is one of the largest and longest available in the literature, to our best knowledge, and demonstrated that visual fatigue did not increase with refractive disorders or with visual acuity with lenses, or in relation to work seniority and age, confirming that symptoms are not harmful for the eye. Visual symptoms are mainly caused by anxiety perception and psychosocial factors: this finding suggests the need to consider this and to suggest interventions to increase wellness in workplaces, but also the need to emphasize the safe characteristic of work with VDT, avoiding the overestimation of symptoms without signs of diseases. Moreover, visual symptoms in general are mild. To confirm this, the majority of workers reported “visual fatigue in relation to VDTs sometimes”, while “visual fatigue in relation to VDTs often” was reported by a lower percentage of workers. Irritant symptoms such as “red eye” and “burning eye” were reported by less than 7% of subjects, meaning that important symptoms were rare, and acute and disabling symptoms were never reported [53]. Moreover, myopia did not increase during the follow-up, confirming what was already demonstrated by Rechichi et al. in 1996 [53] following 23,000 VDT users for 4 years. This author showed no relationship between refractive errors and VDT exposure [48], and a review of Mutti and Zandik [54] concluded that there is no evidence that VDT work causes myopia. Visual fatigue is a symptom and not a sign of eye disease, and Cole in 2003 [55] questioned the need for vision screening in VDT workers. He said that visual discomfort is common among workers even if they do not use VDTs and that visual screening would be better applied for workers when vision is critical for safety, such as professional drivers, air traffic controllers, etc.

## 5. Conclusions

Our study, in a long follow-up, failed to find an increase of refractory disorders in VDT users. Visual fatigue was common and symptoms were related to many factors, but not to refractive disorders and visual acuity with lenses. Anxiety perception was of paramount importance compared to environmental factors, use of lenses, time of VDT use and psychosocial factors. Visual fatigue did not increase in relation to age, and seniority of work and gender did not play any role in the occurrence of symptoms. In our 10-year follow-up, refractive disorders increased mainly in relation to age as presbyopia and hyperopia, while no increase in the prevalence of myopia was found, confirming that VDT use is not harmful for vision.

This study emphasizes the need to promote the wellness of workers, focusing on interventions to reduce anxiety, increasing good relationships between workers, and promoting a good psychosocial environment together with a good control of environmental variables and the correction of refractive disorders, which seems to be not relevant compared to other factors. Effort can be put into the good organization of the workplace, the environmental control of lights and a better evaluation of psychological factors.

## Figures and Tables

**Table 1:** Characteristics of participants, use of lenses and visual symptoms during the follow-up.

Characteristics	Description	1st Examination	2nd Examination	3rd Examination	Last Examination
Men (*n*)		1537	1032	841	1138
Women (*n*)		1558	1176	809	1070
Total (*n*)		3095	2208	1650	2208
Age Years (mean ± SD)		36.7 ± 10.5	41.1 ± 9.1	47.8 ± 8.2	46.5 * ± 9.3
Work seniority years (mean ± SD)		7.4 ±6.7	9.2 ±6.5	13.7 ±7.0	14.5 * ± 7.4
VDT per day hours (mean ± SD)		5.0 ± 2.5	4.8 ±2.1	5.4 ±2.0	5.4 ±1.9
Visual defects	Hyperopia %	7.2	7.5	9.9	11.6 *
	Myopia %	46.8	44.7	45.3	45.1
	Astigmatism %	33.2	37.1	43.0	40.8
	Presbyopia %	22.3	36.8	61.3	52.3 *
Use of lenses	%	80	82	84	82
Visual fatigue	Sometimes %	46.3	48.8	48.4	50.3
	Often %	12.1	13.8	17.3	13.7
Ocular redness	Sometimes %	23.9	26.0	27.6	28.2
	Often %	5.0	7.2	8.5	7.1
Ocular burning	Sometimes %	20.9	22.1	21.9	23.7
	Often %	4.5	6.6	7.6	5.82
Ocular tearing	Sometimes %	11.2	12.4	14.5	15.6
	Often %	2.5	3.4	4.4	4.2
Headache	Sometimes %	9.5	12.2	10.8	11.2
	Often %	2.5	3.5	3.6	2.8
Photophobia	Sometimes %	8.2	10.9	11.4	9.0
	Often %	2.0	2.8	3.4	3.1
Blurry vision far	Sometimes %	10.6	10.5	9.8	9.9
	Often %	2.7	3.0	3.1	3.2
Blurry vision near	Sometimes %	6.8	10.0	11.2	9.8
	Often %	1.9	3.1	3.8	3.1
Myodesmopsia	Sometimes %	7.1	8.3	10.4	9.5
	Often %	1.1	1.4	1.6	1.4
Double vision	Sometimes %	6.2	7.9	9.2	7.0
	Often %	1.5	1.4	2.1	2.1
Eye drop application	Sometimes %	3.8	3.9	6.6	5.4
	Often %	1.2	1.1	1.6	2.0
Anxiety	Sometimes %	2.9	3.7	4.2	3.1
	Often %	0.6	0.9	1.1	1.1

* *p* < 0.005.

**Table 2:** Pearson’s correlation between visual fatigue and environmental variables.

Variables	Local Light	Natural Light	Contrast	Light Flickering	Reflections	Distance	Image
Local light	-						
Natural light	0.17 **	-					
Contrasts	0.22 **	0.31 *	-				
Light flickering	0.15 **	0.02	0.36 **	-			
Reflections	0.27 **	0.01	0.26 **	0.27 **	-		
Distance	0.33 **	0.02	0.25 **	0.19 **	0.24 **	-	
Image	0.29 **	0.02	0.26 **	0.27 **	0.25 **	0.33 **	-
Visual fatigue	0.06 **	0.01	0.05 **	0.03*	0.06 **	0.03 **	0.03 *

* *p* < 0.05; ** *p* < 0.01 (two tails).

**Table 3:** Relationships between visual fatigue and psychosocial variables, analyzed using Pearson’s correlation.

Variables	1	2	3	4	5	6	7	8	9	10	11	12	13	14	15
1 Overcrowding	-														
2 Relationship with users	0.45 **	-													
3 Relationship with collegues	0.30 **	0.42 **	-												
4 Work condition	0.37 **	0.41 **	0.36 **	-											
5 Responsibility	0.26 **	0.39 **	0.39 **	0.33 **	-										
6 Repetitiveness	0.31 **	0.20 **	0.19 **	0.23 **	0.09 **	-									
7 Excessive concentration	0.24 **	0.27 **	0.27 **	0.29 **	0.43 **	0.11 **	-								
8 Excessive attention	0.19 **	0.24 **	0.24 **	0.24 **	0.41 **	0.09 **	0.66 **	-							
9 Low control of mistakes	0.37 **	0.31 **	0.31 **	0.36 **	0.32 **	0.33 **	0.26 **	0.22 **	-						
10 Frequent interruptions	0.34 **	0.29 **	0.26 **	0.28 **	0.27 **	0.27 **	0.22 **	0.19 **	0.40 **	-					
11 Computer slowness	0.28 **	0.25 **	0.19 **	0.25 **	0.14 **	0.30 **	0.16 **	0.10 **	0.36 **	0.45 **	-				
12 Variability	0.29 **	0.37 **	0.33 **	0.33 **	0.43 **	0.11 **	0.36 **	0.29 **	0.32 **	0.29 **	0.18 **	-			
13 Low control	0.35 **	0.33 **	0.32 **	0.34 **	0.31 **	0.32 **	0.24 **	0.23 **	0.38 **	0.32 **	0.24 **	0.30 **	-		
14 Slow cadence	0.28 **	0.25 **	0.21 **	0.22 **	0.12 **	0.43 **	0.09 **	0.05 **	0.31 **	0.28 **	0.34 **	0.14 **	0.28 **	-	
15 Fast cadence	0.28 **	0.22 **	0.21 **	0.24 **	0.33 **	0.14 **	0.40 **	0.41 **	0.22 **	0.24 **	0.12 **	0.36 **	0.31 **	0.04 **	-
16 Visual fatigue	0.04 **	0.02	0.03 **	0.03 *	0.02	0.01	0.05 **	0.04 **	0.01	0.02	0.01	0.03 *	0.04 **	0.00	0.04 **

* *p* < 0.05; ** *p* < 0.01 (two tails).

**Table 4:** Visual fatigue, visual acuity and refractive disorders in the first and in the last control evaluated with univariate logistic regression. Data are reported as odds ratios (OR) and 95% confidence intervals (CI).

Variables	1st Examination			Last Examination		
	OR	95% CI	*p*	OR	95% CI	*p*
Sex female	**1.3**	**1.1–1.56**	**0.005**	0.98	0.81–1.17	0.814
Age	**0.98**	**0.98–0.99**	**0.031**	**0.98**	**0.97–0.99**	**0.001**
Seniority of work	1.00	0.99-1.01	0.753	**0.98**	**0.97–0.99**	**0.022**
VDT working hours	**1.19**	**1.14–1.24**	**0.000**	**1.17**	**1.11–1.23**	**0.000**
Use of lenses	**1.42**	**1.1–1.85**	**0.011**	1.28	0.99–1.64	0.055
Presbyopia	**0.82**	**068–0.98**	**0.033**	0.89	0.74–1.05	0.186
Hyperopia	**1.52**	**1.1–2.2**	**0.020**	0.98	0.75–1.30	0.889
Astigmatism	**1.30**	**1.05–1.52**	**0.012**	1.05	0.88–1.25	0.582
Myopia	1.09	0.91–1.31	0.301	0.93	0.78–1.10	0.415
Visual acuity OO near	1.02	0.98–1.07	0.431	1.01	0.96–1.01	0.628
Visual acuity OO intermediate	1.06	0.96–1.17	0.213	1.02	0.98–1.12	0.137
Visual acuity OO far	1.02	0.92–1.13	0.734	1.10	0.98–1.24	0.112

VDT: video display operators; OO: both eyes; In bold, significant results.

**Table 5:** Factors associated to visual fatigue in the first examination, evaluated with multivariate logistic regression (*n* = 3054 workers).

Variables	Odds Ratio	95% CI	Z Score	*p* Value
Female gender	1.09	0.94–1.28	1.18	0.239
Age	0.99	0.98–1.00	−1.52	0.128
Seniority of work	1.00	0.99–1.01	1.01	0.314
VDT working hours	**1.17**	**1.13–1.22**	**8.79**	**0.000**
Use of lenses	**1.30**	**1.08–1.58**	**2.71**	**0.007**
Presbyopia	0.87	0.67–1.13	−1.02	0.310
Hyperopia	**1.56**	**1.1**–**2.3**	**2.28**	**0.023**
Astigmatism	1.2	0.96–1.4	1.57	0.117
Environmental factors	1.10	0.99–1.30	1.78	0.078
Psychosocial factors	**1.03**	**1.00**–**1.06**	**2.25**	**0.025**
I feel anxious sometimes during VDT use	**2.62**	**1.56**–**4.4**	**3.65**	**0.000**
I feel anxious often during VDT use	**4.97**	**1.48**–**6.84**	**2.59**	**0.010**
I feel anxious not in relation with VDT use	**1.40**	**1.07**–**1.84**	**2.25**	**0.025**

CI: confidence interval; in bold, significant results.

**Table 6:** Factors associated to visual fatigue during 10-year follow-up using the generalized estimating equation (2208 workers that repeated medical examinations 3 times were included in the analysis).

Variables	Odds Ratio	95% CI	Z Score	*p* Value
Female gender	1.05	0.90–1.24	0.66	0.508
Age	**0.98**	**0.97**–**0.99**	**−2.5**	**0.015**
Seniority of work	1.02	0.88–1.35	0.65	0.450
VDT work hours	**1.27**	**1.04**–**1.53**	**2.50**	**0.035**
Use of lenses	**1.34**	**1.09**–**1.64**	**2.80**	**0.005**
Environmental factors	**1.02**	**1.01**–**1.06**	**2.15**	**0.025**
Psychosocial factors	**1.03**	**1.01**–**1.07**	**2.23**	**0.026**
I sometimes feel anxious during VDT use	**2.69**	**1.57**–**4.61**	**3.60**	**0.000**
I often feel anxious during VDT use	**7.40**	**1.77**–**31.03**	**2.74**	**0.000**
I do not feel anxious in relation to VDT use	1.01	0.78–1.30	0.13	0.944

CI: confidence interval; in bold, significant results.
